# Native Aortic and Tricuspid Valve Endocarditis Complicated by Embolic ST Elevation Myocardial Infarction

**DOI:** 10.1155/2019/1348607

**Published:** 2019-03-03

**Authors:** Mumtaz Zaman, Richard Loynd, Anthony Donato

**Affiliations:** ^1^Sidney Kimmel Medical College at Thomas Jefferson University, USA; ^2^Tower Health System, USA

## Abstract

Acute myocardial infarction due to a coronary embolic event can occur as a complication of infective endocarditis in up to 2.9% of cases and can frequently be the presenting symptom. A 35-year-old female presented with 4 hours of typical chest pain and was found to have ST elevations in inferior leads as well as an elevated serum Troponin I of 8.29 ng/ml (normal: <0.06 ng/ml). Urgent cardiac catheterization revealed total occlusion of the right coronary artery without other coronary disease or collaterals. Following a failed attempt at thrombus extraction, a 3.0 × 38 mm bioabsorbable drug-eluting stent was placed. Echocardiography then revealed large mobile aortic valve vegetations with the largest measuring 1.4 × 1.7 cm, severe tricuspid regurgitation with a 1.1 × 0.5 cm mobile vegetation on the anterior leaflet along with a patent foramen ovale with right-to-left shunting. Blood cultures identified *Enterococcus faecalis* in 4 of 4 vials. The patient underwent urgent replacement of tricuspid and aortic valves as well as 6 weeks of IV antibiotics followed by chronic antibiotic suppression.

## 1. Introduction

Endocarditis continues to be the fourth most common life-threatening infection, accounting for 1.58 million disability life-years lost globally [1]. While symptoms of bacteremia, peripheral embolization, and vascular phenomena are the classic symptoms of endocarditis, cardiac embolic disease has been reported in up to 60% of patients in autopsy studies [2] and may present antemortem as acute myocardial infarction (AMI) in up to 2.9% [3]. Recognition of AMI secondary to embolic disease is critical, as it affects decisions on both pharmacologic and mechanical interventions.

## 2. Case

**Figure 1:**
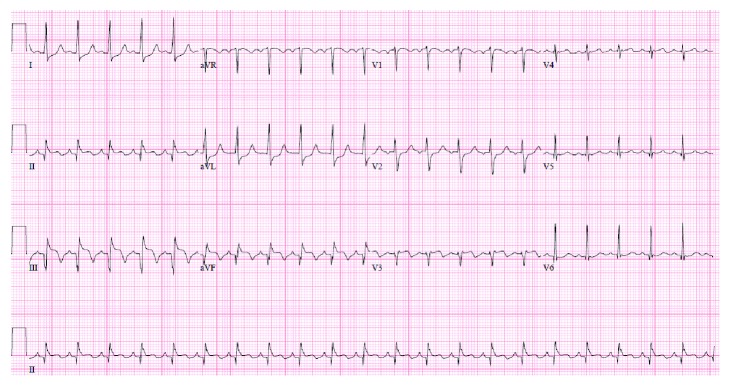
Electrocardiogram on admission showed inferior wall ST elevation myocardial infarction. ST elevations in leads II, III, and aVF.

**Figure 2:**
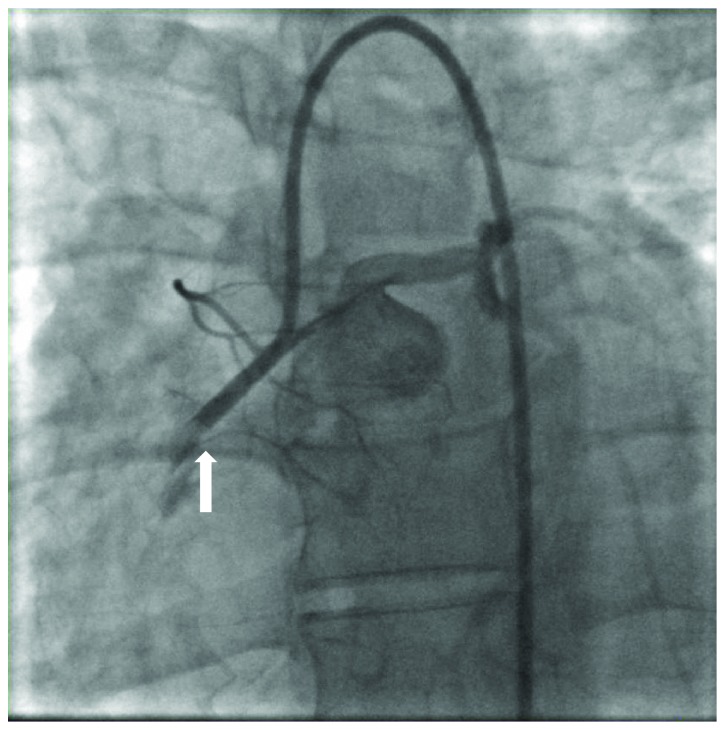
Coronary angiography with 100% occlusion (arrow) at the midsegment of the right coronary artery from infective endocarditis embolus.

**Figure 3:**
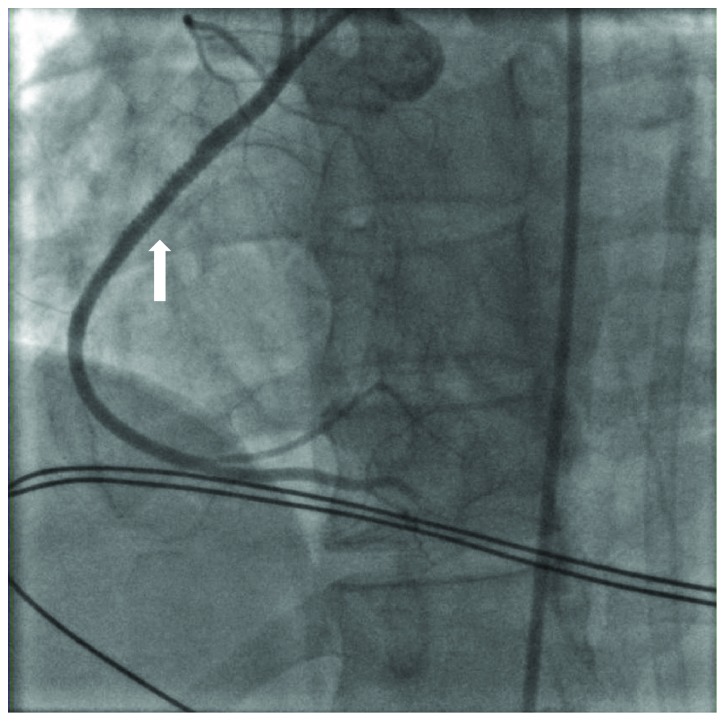
Coronary angiography after synergy 3.0 × 38 mm drug-eluting stent (arrow) placed in RCA with return of TIMI 3 flow.

**Figure 4:**
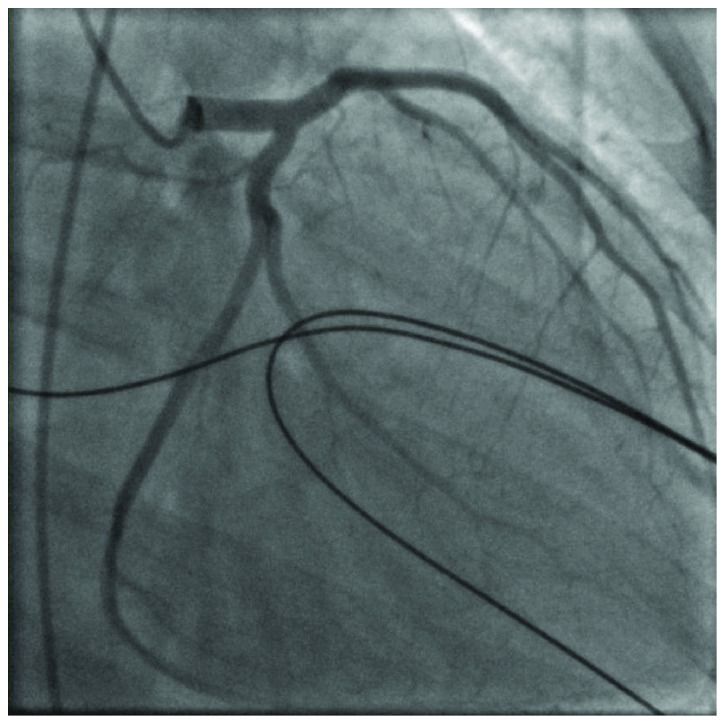
Coronary angiography showing widely patent left main, LAD, and circumflex, without evidence of chronic atherosclerosis.

**Figure 5:**
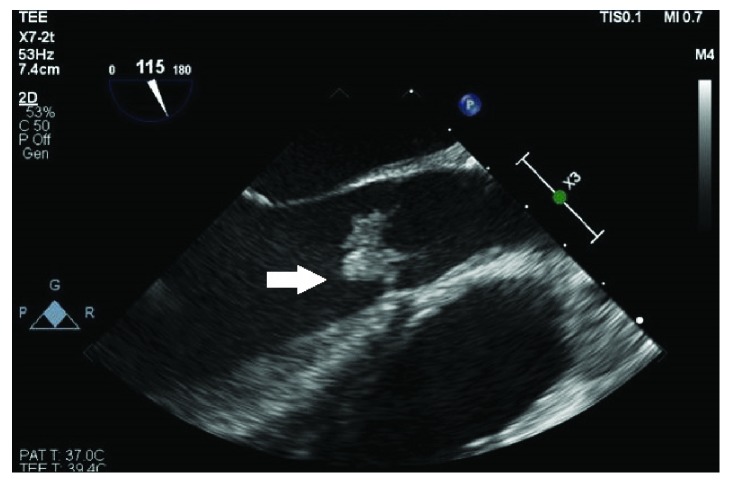
Transesophageal echocardiography revealing large aortic vegetation in longitudinal view (arrow).

**Figure 6:**
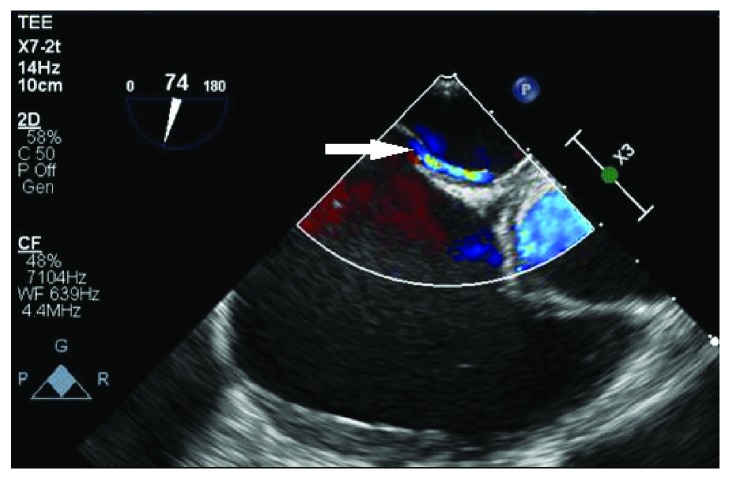
Transesophageal echo showing patent foramen ovale (arrow).

A 35-year-old female with a past medical history of type 1 diabetes mellitus, hypertension, and hyperlipidemia presented to emergency care with 4 hours of continuous sharp midsternal chest pain. At the time of admission, she admitted to intermittent issues with hyperglycemia over the last several weeks as well as night sweats and malaise but denied fever, weight loss, cough, nausea, vomiting, abdominal pain, dysuria, or vaginal discharge. There was no history of recent travel, cardiac abnormalities, dental procedures, or exposure to tobacco, alcohol, or illicit drugs. Surgical history was pertinent for a Cesarean section performed 3 months prior to admission complicated by postpartum hemorrhage from placental abruption. On exam, she was awake and alert with no respiratory distress. Her vital signs were BP 116/72 mmHg, pulse 97 bpm, temperature 99.7°F, and respiratory rate 24 breaths/minute on room air. The physical examination was normal except for a systolic ejection murmur at the left lower sternal border and a well-healed low transverse Cesarean section incision. Her lab data showed a WBC count of 10,500 c/mm^3^ (normal: 4,800–10,800 c/mm^3^), hemoglobin of 7.6 g/dl (normal: 12.0–16.0 g/dl), MCV 84.5 fl (normal: 80.0–9.0 fl), creatinine of 1.58 mg/dl (normal: 0.60–1.30 mg/dl), and Troponin I of 8.29 ng/ml (normal: <0.03 ng/ml). EKG demonstrated ST elevations in leads II, III, and aVF consistent with inferior acute myocardial infarction (Figure 1). She was taken emergently to the cardiac catheterization lab where she was noted to have a completely occluded right coronary artery (RCA) (Figure 2). Consistency of the occlusion and absence of calcium suggested acute thrombus or embolus. An aspiration was attempted but was unsuccessful, so a 3.0 × 38 mm bioabsorbable drug-eluting stent was placed into the RCA along with balloon dilation of the proximal aspects of the posterior descending artery and distal RCA (Figure 3). Left-sided coronaries were angiographically normal (Figure 4). A postcatheterization transthoracic echocardiogram then revealed large mobile aortic valve vegetations on all 3 leaflets with the largest measuring 1.4 × 1.7 cm, moderate aortic insufficiency, severe tricuspid regurgitation with a 1.1 × 0.5 cm mobile vegetation on the anterior leaflet of tricuspid valve, and moderate mitral regurgitation without lesions. Ejection fraction was estimated at 50% with inferior and inferolateral wall hypo/akinesis. These findings were later confirmed with transesophageal echocardiogram, which also identified a patent foramen ovale with continuous right-to-left shunt (Figures 5 and 6). Chest CT imaging noted no embolic disease.

Blood cultures grew *Enterococcus faecalis* in 4 of 4 vials. Vancomycin and ceftriaxone were initiated and the patient was transferred to an outside facility where she underwent urgent cardiac surgery with the placement of bioprosthetic aortic and tricuspid valves, as well as PFO closure. Bioprosthetic valves were used as per patient preference. Cultures of removed cardiac tissue were positive for *Enterococcus faecalis*. No evidence of aortic root abscess was noted during the surgery. CT of the chest, abdomen, and pelvis was unrevealing for sepsis source. Endocarditis was ultimately attributed to prior placental abruption.

After valve replacement, she was continued on IV antibiotics. Blood cultures cleared, and on hospital day 15, she was discharged on IV ampicillin and ceftriaxone for synergy. Three weeks after discharge, a repeat ECHO revealed an ejection fraction of 47% and severe hypokinesis of the basal to mid inferior wall with normal function of both aortic and tricuspid bioprosthetic valves. After completing 6 weeks of IV antibiotic therapy, she was transitioned to chronic oral suppression with amoxicillin/clavulanic acid. At phone follow-up 6 months later, the patient reported tolerating oral suppressive antibiotic with no clinical signs or symptoms of recurrent infection or complications.

## 3. Discussion

While most acute myocardial infarctions are secondary to atherosclerotic heart disease, the differential also includes less common nonatherosclerotic causes. These include vasculitis, congenital coronary artery abnormalities, vasospasm, coronary embolism, and coronary artery extrinsic compression by either abscess or pseudoaneurysm [2, 4]. Since the initial description of coronary emboli by Dr. Virchow in 1856 [5], embolic infarction due to infective endocarditis (IE) has remained an underrecognized entity. Sudden onset embolic infarctions are believed to occur in 2.9% of all cases of infective endocarditis [3] and are associated with very high rates of subsequent heart failure (73%) [4] and death (64%) [3].

Presentation with embolic AMI typically occurs within 2 weeks of a known diagnosis of endocarditis [6] but can be the initial presenting symptom in up to 54% of cases [4]. Identified risks for AMI in IE include larger (>1 cm) vegetations, mobile vegetations, and the presence of a valve ring aortic abscess [3, 7]. However, both aortic and mitral valves are associated with AMI. Our case was unusual in that we identified a tricuspid valve vegetation and a PFO with right-to-left shunting without pulmonary infarction or emboli. This suggests that the tricuspid valve was likely infected first, then bypassing the pulmonary circulation through the PFO, led to the infection of the aortic valve, which has only been suspected once before [8].

Clinical findings at cardiac catheterization that suggest embolic disease include abrupt and complete occlusion in a single coronary artery, especially in the absence of other coronary plaques and the absence of collateral blood flow. The management of these infected emboli remains unclear. Thrombolytics seem to release previously walled-off vegetations on valves, resulting in cerebral embolic showers followed by serious intracranial bleeds and the worsening of infection in both animal models [9] and in human case reports [10] and are therefore not recommended. Catheter-based therapies such as angioplasty, PCI, and embolectomy have also been used. Angioplasty is often immediately unsuccessful due to the lack of plaque to expand the vessel lumen, leading to rapid reocclusion [11]. PCI has been shown to be more effective immediately; however, it carries the risk of stent infection and, along with angioplasty, the risk of mycotic aneurysm with ultimate concern for coronary artery rupture and sudden death [12]. Per ACC guidelines, valve replacement is recommended in patients with recurrent emboli (Class IIa recommendation) or heart failure (Class I indication) [13].

While the diagnosis of AMI due to embolic IE remains rare, it is important to consider as treatment and clinical course vary significantly from atherosclerotic AMI. When presented with a young patient without typical risk factors for coronary heart disease, with early fevers, or history of endocarditis, physicians should consider an urgent TEE for evaluation of IE [3], which has a sensitivity of 93% for detecting valvular vegetations [14]. Once diagnosed, we endorse a multidisciplinary approach [4] and propose systemic antibiotics with or without emergent PCI as a bridge to possible surgical valve replacement. Further studies are recommended to help guide future therapy options.
